# Recent advances in transducers for through-tissue ultrasonic power transfer

**DOI:** 10.1088/2516-1091/ae5f8a

**Published:** 2026-05-07

**Authors:** Yu Chu, Ali Naderi, Huaiyu Wu, Xiangming Xue, Michael L Oelze, Xiaoning Jiang

**Affiliations:** 1 Department of Mechanical and Aerospace Engineering, North Carolina State University, Raleigh, NC 27606, United States of America; 2 Joint Department of Biomedical Engineering, North Carolina State University, Raleigh, NC 27695, United States of America; 3 Department of Electrical and Computer Engineering, University of Illinois at Urbana-Champaign, Urbana, IL 61801, United States of America

**Keywords:** ultrasonic power transfer, wireless energy transmission, transducer architectures, piezoelectric, PMUT, CMUT, implantable and wearable devices

## Abstract

Ultrasonic power transfer (UPT) is gaining traction for wireless energy delivery to implants and wearables because it combines centimeter-scale penetration with compact receivers. This review takes a transducer-centric view of UPT and organizes the field across bulk piezoelectrics (including lead-free options), piezoelectric micromachined ultrasonic transducers, capacitive micromachined ultrasonic transducers, flexible polymer platforms and magnetostrictive transducers. We connect working mechanisms and structural configurations to practical performance—operating frequency ranges, bandwidth, link efficiency and output power, and miniaturization trade-offs—and summarize representative demonstrations in biomedical systems. System-level considerations for integration (acoustic/electrical matching and rectification) and bidirectional links (including backscatter and active telemetry) are highlighted to show how a single acoustic carrier can deliver power and data through tissue. We conclude with challenges (attenuation and misalignment, materials reliability and packaging, and scaling to millimeter/sub-millimeter form factors) and opportunities that draw on materials innovations (metamaterials, lead-free ceramics, flexible polymers) and machine-learning-assisted co-design for robust, efficient through-tissue operation. Together, this transducer-focused synthesis provides a practical map from device physics and fabrication choices to system performance and emerging applications.

## 1. Introduction

Ultrasonic power transfer (UPT) has attracted growing interest as a promising method for wireless energy delivery across air, metal, and biological tissues [1]. As implantable and wearable devices continue to shrink while expanding in functionality, reliable wireless power delivery across biological tissue has become a central challenge. In the broader wireless power transfer (WPT) landscape—including magnetic inductive/resonant links, capacitive coupling, far-field RF/microwave transmission, optical/IR beaming, and magnetoelectric hybrids—each approach offers distinct advantages but also faces intrinsic limitations for deep-tissue applications, such as short-range efficiency and tight alignment (inductive/capacitive), specific absorption rate-limited power and tissue attenuation (RF), line-of-sight constraints and scattering (optical), and bias-field or alignment complexity (magnetoelectrics).

Within this context, UPT exploits the MHz acoustic window of soft tissue and couples efficiently to ultrasonic receivers [2]. In tissues, focused ultrasound can reach depths on the order of several to tens of centimeters while providing high spatial selectivity, as demonstrated in clinical focused-ultrasound systems [3], and beamforming theory shows that electronic focusing/steering can offer some tolerance to moderate misalignment [4]. Recent UPT links have demonstrated that a single ultrasonic carrier can deliver power while supporting data telemetry via passive backscatter modulation [5] or active uplink transmission [6]. Because the carrier is mechanical rather than electromagnetic, ultrasonic links are inherently immune to electromagnetic interference and can operate in conductive liquids or metallic environments where RF coupling is degraded [7]. Critically, UPT can meet medical safety limits and still deliver *µ*W–mW level power, enabling miniaturized, long-lived, and wirelessly recharged for implantable medical devices (IMDs). Khan *et al* [8] review WPT strategies for implants and identify ultrasound as particularly suitable for deep-tissue operation, while Zheng *et al* [9] analyze recent advances in UPT efficiency, acoustics, and safety.

The physical suitability of ultrasound for through-tissue energy delivery is rooted in its long-standing use in medicine. In 1942, Dr Karl Theodore Dussik reported the first demonstration of medical ultrasonic transmission through the brain [10], marking the beginning of clinical ultrasonics. Although subsequent decades saw extensive development of ultrasound for imaging and therapy, the concept of using ultrasonic waves for transcutaneous energy transfer did not re-emerge until the early 2000s. A landmark study in 2009 demonstrated the feasibility of ultrasonic transcutaneous energy transfer, achieving a peak power transfer efficiency of 27% and an output power of 70 mW using lead zirconate titanate (PZT)-based transducers operating at 673 kHz, thereby validating UPT as a practical approach for powering implanted devices requiring deep penetration and minimal invasiveness [11].

Building on this milestone, recent research has increasingly focused on overcoming practical constraints in real-world deployment. Advances in transducer technologies have played a central role in this evolution. The development of flexible ultrasonic transducers has enabled improved conformity to curved biological surfaces and enhanced biocompatibility [12, 13], while the integration of micromachined ultrasonic transducers (MUTs), including CMUTs and PMUTs, has facilitated system miniaturization and efficient power delivery in implantable and wearable platforms [14]. Recent reviews further consolidate device architectures, fabrication routes, and biomedical integration trends for PMUT/CMUT platforms, providing a useful perspective on performance trade-offs relevant to UPT miniaturization and packaging [15]. In parallel, emerging studies have explored acoustic metamaterials to enhance UPT efficiency in challenging environments, such as transmission through metallic barriers, further broadening the applicability of UPT systems [16].

These transducer and system-level advances have enabled a growing range of biomedical applications, including neural stimulation, drug delivery, and biosensing, reflecting a shift from single-function power links toward multifunctional and versatile UPT platforms [17, 18].

Motivated by the rapid evolution of UPT technologies, this review provides a transducer-centric synthesis of through-tissue UPT. We examine how transducer materials, architectures, and operating principles govern key performance trade-offs, and we discuss emerging strategies aimed at improving efficiency, miniaturization, and robustness for practical biomedical and industrial applications.

## 2. Principles of ultrasonic power transmission

### 2.1. Mechanism of UPT

**Figure 1.**
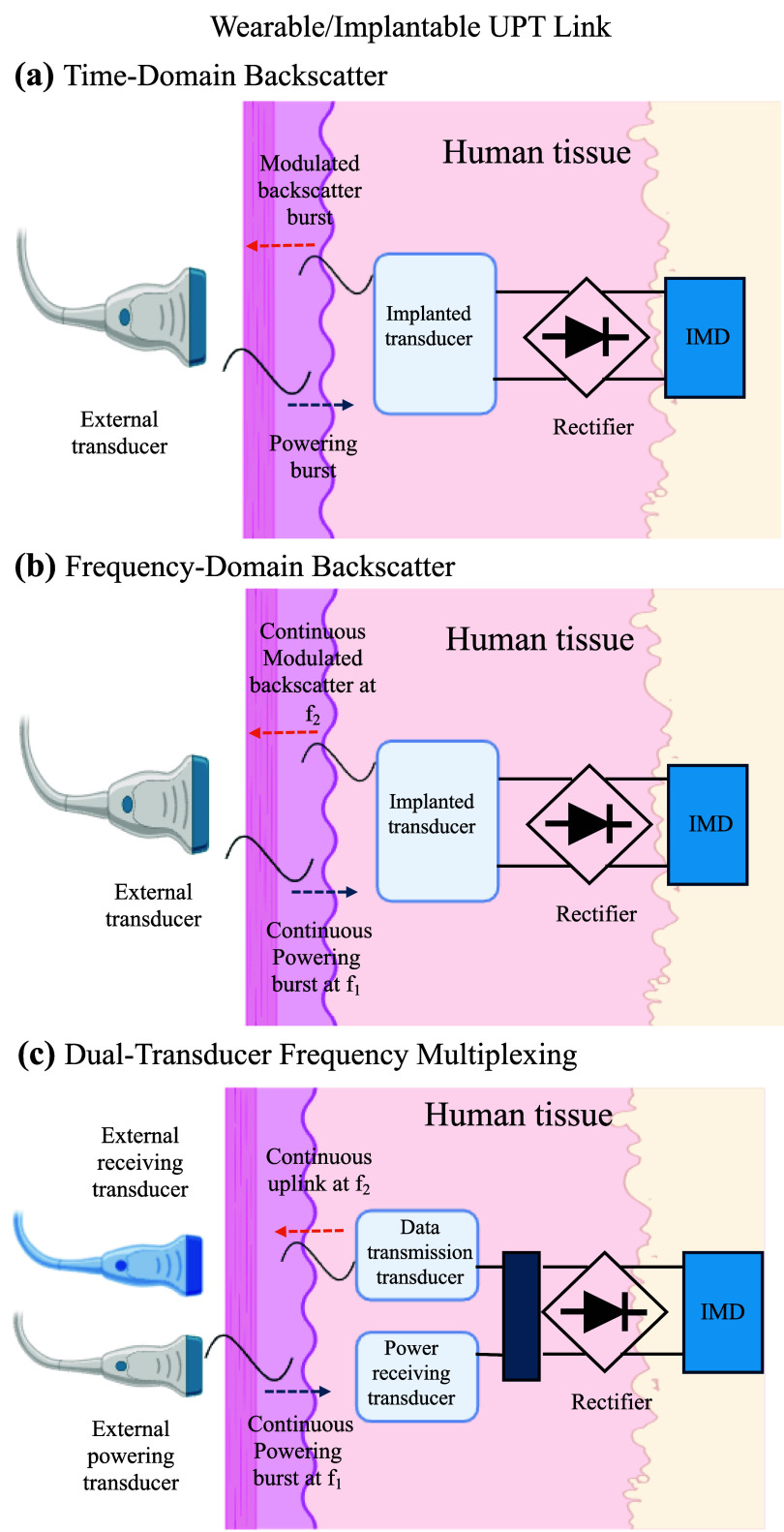
Ultrasonic power transfer (UPT) architectures for combined wireless power delivery and data communication: (a) time-domain backscatter modulation. (b) Frequency-domain backscatter modulation. (c) Dual-transducer frequency-multiplexed link.

The fundamental operation of UPT in biomedical applications can be illustrated through a typical system architecture for implantable or wearable devices. In such systems, an external acoustic transmitter delivers ultrasonic energy through biological tissue to an implanted or surface-mounted receiver. This end-to-end architecture of wearable and implantable UPT designs is schematically summarized in figure 1, which highlights three fully ultrasonic strategies that simultaneously deliver power and transmit data. Figure 1(a) depicts time-domain backscatter modulation, where the implant alternates between harvesting acoustic power and reflecting it for communication. Figure 1(b) shows frequency-domain backscatter modulation, which maintains continuous powering while encoding data as spectral sidebands, enabling uninterrupted operation. Figure 1(c) illustrates a dual-transducer frequency-multiplexed configuration in which one transducer is optimized for power reception and another actively transmits data on a different frequency. Among these, frequency-domain backscatter (figure 1(b)) enables continuous, simultaneous power and data transfer and has been demonstrated for deep implants using robust frequency-domain backscatter modulation [19]. Time-domain backscatter (figure 1(a)) is limited to very low data-rate uplinks and, as a passive scheme, benefits from knowing the implant’s location *a priori* for reliable *in-vivo* detection; the dual-transducer approach (figure 1(c)) increases implant size and circuit complexity. To improve *in-vivo* detectability when implant localization is uncertain, some systems actively re-emit ultrasound—trading extra circuit complexity for robust visibility during targeting and readout.

By adding an energy-storage element, a receiver can later operate as a transmitter after sufficient charge has accumulated. Once a defined threshold is reached, the implant can emit ultrasound (or electrical stimulation (ES)), enabling either localization/tracking or targeted therapeutic intervention. Externally, a single-element probe or phased array can detect these emissions using imaging-based methods to support closed-loop targeting and bidirectional communication. This concept has been demonstrated in an ultrasound-identifiable radiological clip for improved surgical localization [20].

Some UPT links can also reverse the power flow and transmit acoustic energy from the implant to the exterior. In this ‘inside-out’ mode, the implant stores incident energy in a capacitor or micro-battery and later emits a controlled ultrasonic pulse. The returned wave can deliver power (e.g. to recharge a wearable relay) and/or convey information such as physiological signals or device status. This capability supports self-diagnosis and closed-loop feedback without adding separate RF or optical links. Recent work has demonstrated mm-scale UPT receivers that support both ultrasonic powering and an acoustic uplink [5, 21]. Such systems can deliver short ‘power-out’ bursts while operating within FDA-safe acoustic intensity limits [19]. Such capabilities expand UPT from one-way energy delivery into a full bidirectional energy–data network, supporting autonomous implants, adaptive therapies, and distributed intrabody power sharing.

From a systems perspective, UPT enables robust wireless energy delivery in confined or electromagnetically challenging environments. Nonetheless, UPT links face two recurring limitations: acoustic attenuation along the propagation path, which reduces delivered acoustic energy [22], and sensitivity to transmitter–receiver alignment, which degrades acoustic coupling and transfer efficiency [23]. In addition, small passive implants can be difficult to visualize *in vivo*, complicating probe-to-implant alignment during powering. These factors significantly impact the performance and practicality of UPT systems, particularly in biomedical applications where energy must penetrate biological tissues effectively while minimizing losses and ensuring patient safety.

### 2.2. Key specifications of UPT systems

The performance of UPT systems is accessed using several key evaluation parameters that directly impact their efficiency and reliability. The receiver, usually integrated with rectification, regulation, and control electronics,—converts the received acoustic energy into a stable electrical supply for powering embedded circuits such as sensors, stimulators, or therapeutic devices. UPT waves typically operate at frequencies ranging from 20 kHz to several MHz, with the specific operating range selected based on the target medium and application (table 1) [21]. The selection of the appropriate frequency is critical, as it affects not only the depth of penetration but also the energy transfer efficiency and spatial alignment between the transmitter and receiver in the system. Higher frequencies generally offer superior resolution and enable the miniaturization of devices, which is advantageous for biomedical applications such as IMDs [24]. However, they come at the cost of greater attenuation, particularly when propagating through dense or lossy environments like biological tissues [25]. Attenuation itself is a key parameter, representing the progressive loss of ultrasonic wave energy as it traverses a medium. This phenomenon is shaped by factors such as medium density, acoustic impedance, and wave frequency. In applications involving complex media—like human tissues—minimizing attenuation is crucial to ensure that sufficient power reaches the implanted or embedded device [22]. Furthermore, strategies like impedance matching and optimized wave focusing have been explored to combat energy losses due to attenuation [26].

**Table 1.** Frequency ranges and corresponding applications in UPT, illustrating optimal frequency selection for industrial sensing, medical implants, wearable devices, and micro-scale energy harvesting.

Frequency range	Key applications	Transducer types	References
20 kHz–100 kHz	Industrial sensors, underwater systems	Piezoelectric (PZT), Magnetostrictive, electromagnetic acoustic transducers (EMATs)	[30, 31]
100 kHz–1 MHz	Robotics, remote industrial systems	CMUTs, piezoelectric arrays, air-coupled transducers	[32, 33]
1 MHz–3 MHz	Implantable medical devices (pacemakers, implantable micro-optical gyroscopes (IMOG))	PMUTs, flexible polymer transducers, quartz transducers	[34, 35]
3 MHz–10 MHz	Miniaturized implants (retinal/cochlear devices)	CMUTs, lead-free piezoelectrics, MEMS piezoelectrics	[36]
10 MHz–50 MHz	Medical imaging, micro-energy harvesters	MEMS-based nanogenerators, Thin-film piezoelectrics	[37]

As illustrated in table 1, different frequency ranges align with specific application domains [27, 28]. Lower frequencies, typically between 20 kHz and 100 kHz, are well-suited for industrial uses such as underwater communication and structural health monitoring, where deep penetration is essential [29]. In contrast, mid-range frequencies (1–3 MHz) are often selected for IMDs, providing an optimal balance between penetration depth and energy transfer efficiency through biological tissues. Higher-frequency ultrasound (10–50 MHz) is commonly used in medical imaging, where shorter wavelengths improve spatial resolution [29]. Similar frequency ranges are also used in microscale ultrasound energy harvesters, where shorter wavelengths support miniaturized resonators [27].

Beyond attenuation and frequency, parameters such as power density and intensity are equally vital. These metrics quantify the energy delivered to the receiver and are particularly significant in power-hungry applications like neural stimulators or drug delivery systems. Likewise, reflections and refractions at material interfaces can cause substantial energy dispersion, requiring careful management of wave paths and alignment between transducers to maximize energy coupling [38].

In UPT systems, performance metrics such as efficiency, output power, and bandwidth must be interpreted with respect to clearly defined system boundaries. Efficiency, in particular, can refer to different physical processes, including electro–acoustic conversion at the transmitter, acousto–electric conversion at the receiver, or end-to-end link efficiency from electrical input to electrical output. These quantities are fundamentally distinct and depend strongly on impedance matching, acoustic loading, backing design, radiation losses, and biasing conditions. Consequently, intrinsic material or device parameters—such as the electromechanical coupling factor $k$ (or $k_t^2$)—are better interpreted as indicators of transduction capability rather than direct measures of system-level efficiency.

Unlike diagnostic ultrasound imaging, UPT is commonly operated using continuous-wave or narrowband burst excitation, where energy delivery efficiency is prioritized over short pulse duration. As a result, bandwidth is not a defining requirement for power delivery itself, although it can influence alignment tolerance, robustness to frequency drift, and the feasibility of simultaneous power and data transmission [39]. Similarly, achievable output power cannot be treated as an intrinsic technology-level parameter, as it depends strongly on transducer area, allowable drive voltage, acoustic loading, and system configuration.

In light of these considerations, table 2 provides a design-oriented comparison of ultrasonic transducer technologies for power transfer, focusing on transduction characteristics, biasing requirements, and bandwidth capability.

**Table 2.** Summary of performance metrics for various UPT transducer types.

Technology	Transduction metric	Bias requirement	Fractional BW (−3 dB, %)	Peak-to-peak pressure (NF, MPa)	Tx sensitivity (NF, MPa/100 V)	Rx sensitivity (PE, V MPa^−1^)	UPT-relevant notes	References
Bulk PZT (piezoelectric ceramic)	$k_t^2$ ≈ 0.20–0.36 (mode/material dependent)	No DC bias (voltage–driven)	Design-dependent; can be high (e.g. up to ∼90% −6 dB fractional bandwidth has been reported for PMN-PT/epoxy 1–3 composite annular array transducers) [40]	Highly design- and drive-dependent; can reach high pressures (incl. HIFU)	High (geometry & matching dependent)	High (load & matching dependent)	Strong electromechanical coupling, but system electro–acoustic efficiency depends heavily on backing, matching, and impedance (backside loss & mismatch can dominate).	[41]

CMUT	$k_t^2$ ≈ 0.02–0.26 (benchmark; bias-dependent)	DC bias typically ∼70–160 V (benchmark; device/operating-point dependent)	≈25–135 (LF tends higher; HF tends lower)	≈0.2–1.5	≈0.4–1.7	≈3.5–10	High receive sensitivity and broad BW; pressure and Tx sensitivity improve with maturity and operating point; bias voltage and HV electronics can complicate wearable/implant safety & integration.	[42, 43]

Collapsed-CMUT	$k_t^2$ ≈ 0.17–0.27	High DC bias	≈75–115	≈1.7–2.3	≈1.7–2.2	≈6–8	Shows highest peak pressures among CMUT variants; strong candidate where pressure is priority, but still carries HV bias/integration constraints.	[43]
AlN PMUT	$k_t^2$ ≈ 0.01–0.04	No DC bias	≈30–90 (wide variation across samples)	≈0.1–0.2	≈0.1–0.3	≈0.3–3.2	Lower pressure/Tx (maturity-limited), but low dielectric constant can support higher receive sensitivity relative to PZT-PMUT in the benchmark discussion.	

ScAlN PMUT	$k_t^2$ ≈ 0.04–0.08	No DC bias	≈ ∼ 80	≈0.3–0.4	≈ ∼ 0.35	≈1.2–1.6	Sc-doping improves AlN PMUT transduction; still moderate pressure vs CMUT/collapsed-CMUT and PZT-PMUT samples.	[43]

PZT-PMUT	$k_t^2$ ≈ 0.10–0.17	Small DC bias <50 V	≈25–105	≈0.5–2.1	≈0.9–3.6	≈0.2–1.0	Benchmark indicates high pressure/Tx and very low 2nd harmonic (good for controlled high-output operation), but high capacitance can burden drivers and reduce Rx.	[43]

Polymer PMUT	$k_t^2$ ≈ ∼ 0.005–0.01	Small DC bias <50 V	≈ ∼ 25	Very low (near zero)	Very low (near zero)	Very low (near zero)	Polymer-PMUT exhibits very low acoustic output/sensitivity; may suit ultra-low power/low-output niches rather than power transfer.	[43]

## 3. UPT transmitters and receivers

### 3.1. Mechanism-based classification of UPT transducers

Ultrasound wireless power transmission systems rely on transducer technologies whose operating mechanisms, material platforms, and electromechanical characteristics fundamentally determine their suitability as transmitters (TX), receivers (RX), or dual-mode (TX/RX) devices. From both a physical and system perspective, UPT transducers can be broadly classified into five representative categories: bulk piezoelectric transducers, magnetostrictive transducers, capacitive MUTs (CMUTs), flexible polymer-based ultrasonic transducers, and piezoelectric MUTs (PMUTs).

Bulk piezoelectric transducers operate based on the direct *and* inverse piezoelectric effects, where applied electric fields induce mechanical strain (TX operation) and incident acoustic pressure generates electrical charge (RX operation). PZT and related ceramics exhibit high electromechanical coupling coefficients and acoustic power density, making them particularly well suited for high-efficiency power transmission and energy harvesting in UPT systems. Their reversible electromechanical behavior also enables dual-mode TX/RX operation. For example, a single piezoelectric transducer can be time-multiplexed between ultrasonic power reception and telemetry, enabling compact bidirectional links in mm-scale implants [44]. Bidirectional ultrasonic powering with active data communication has also been demonstrated in mm-scale neural-interface platforms [45].

CMUTs generate and detect ultrasound through the vibration of micromachined capacitive membranes driven by electrostatic forces. An applied bias voltage and AC excitation cause membrane deflection and acoustic emission, while incoming acoustic waves modulate the capacitance to produce electrical signals. CMUTs offer excellent miniaturization, broadband response, and CMOS compatibility, making them attractive for receiving and dual-mode TX/RX roles in UPT, especially at high frequencies and in densely integrated implantable systems. However, their required DC bias and associated interface electronics can be a practical constraint for some compact in-body receivers. Their limited output pressure also constrains their use as standalone high-power transmitters [39, 46]. Magnetostrictive transducers rely on the magnetostrictive effect, in which magnetic field variations induce mechanical deformation of ferromagnetic materials such as Terfenol-D. When driven by alternating magnetic fields, these materials generate ultrasonic vibrations with high force output and mechanical robustness. Due to their relatively low electromechanical efficiency and the requirement for external magnetic biasing, magnetostrictive devices are primarily used as high-power ultrasonic transmitters rather than receivers in UPT systems, particularly in industrial or harsh-environment applications [47, 48]. Flexible polymer-based ultrasonic transducers typically employ piezoelectric polymers such as polyvinylidene fluoride (PVDF) and its copolymers. These materials generate electrical charge under mechanical deformation and can be fabricated as thin, flexible films. Their operating mechanism is identical to that of ceramic piezoelectrics, but with significantly lower elastic modulus and acoustic impedance. As a result, polymer transducers offer superior mechanical conformability and biocompatibility, making them particularly suitable for receiving and low-power energy harvesting in wearable and epidermal UPT applications, albeit with reduced conversion efficiency and power handling capability [49, 50].

PMUTs combine thin-film piezoelectric materials (e.g. AlN, ScAlN, or PZT) with micromachined membrane structures that vibrate in flexural modes. In contrast to CMUTs, PMUTs rely on piezoelectric actuation rather than electrostatic forces, enabling lower driving voltages and simplified electronics. Their MEMS-based architecture supports extreme miniaturization and array integration, making PMUTs attractive for highly integrated, size- and power-constrained MEMS implementations, including low-power TX/RX operation and receive functions in specific miniaturized platforms. However, for receive-only implantable links where maximum sensitivity and broad bandwidth are primary requirements, bulk piezoelectric receivers are commonly used. In addition, because PMUTs actuate a flexural membrane rather than a thickness-mode bulk element, their achievable acoustic output pressure/power is typically lower than that of bulk piezoelectric transmitters, which further limits their use as primary transmitters in high-power UPT links [51].

Based on these mechanisms and characteristics, each transducer technology can be categorized according to its dominant functional role in UPT systems. Magnetostrictive devices are generally favored for ultrasonic transmission; bulk piezoelectric receivers are widely used for receiving and dual-mode operation, while CMUTs are also used for receive/dual-mode operation but require DC bias and interface electronics; PMUTs are most commonly used when extreme miniaturization and MEMS/CMOS integration are key constraints; and flexible polymer transducers are primarily used for conformal energy harvesting. Table 2 summarizes this classification and provides a system-level framework for the detailed discussions in section 3.2.

### 3.2. Functional roles of UPT transducers

UPT relies fundamentally on specialized transducers that perform essential roles in generating, propagating, and converting ultrasonic waves to and from electrical energy. These transducers are typically categorized as transmitters, receivers, or dual-mode transducers capable of performing both functions. Each category has distinct operational requirements, material considerations, and design trade-offs, making them suitable for various applications ranging from biomedical implants to industrial monitoring systems. This section provides a comprehensive discussion of these transducer categories, including their underlying physical principles, advantages, limitations, and typical applications, summarized systematically in dedicated tables.

**Figure 2.**
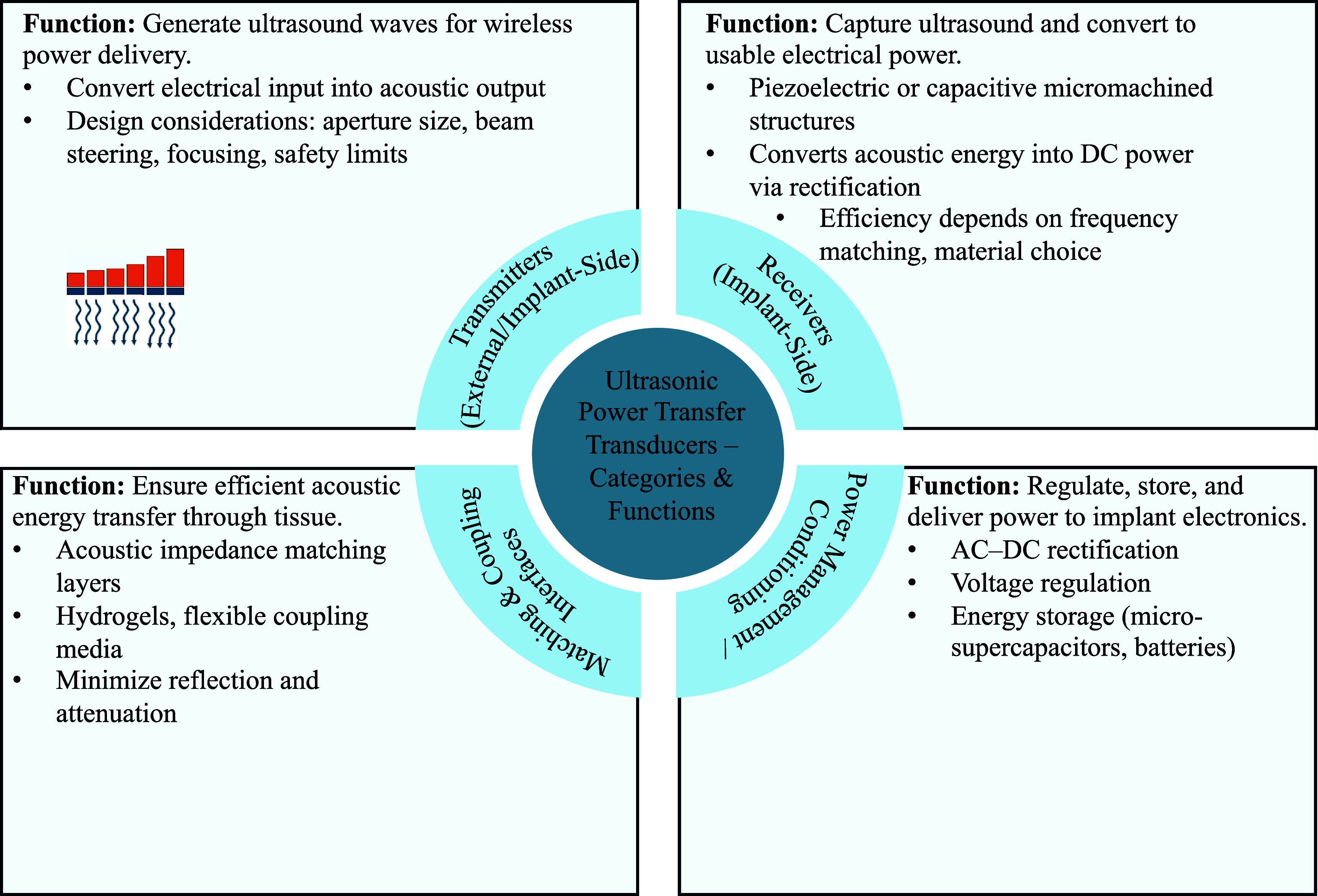
Categories and key functions of UPT transducers. The schematic highlights how UPT systems integrate, (i) transmitters to convert electrical input into acoustic waves, (ii) matching and coupling interfaces to minimize reflection and attenuation during tissue transmission, (iii) receivers to convert acoustic energy back into electrical power, and (iv) power management/conditioning modules to regulate, store, and deliver power to implant electronics.

To provide a system-level overview before delving into each category, figure 2 summarizes the four major functional blocks of UPT transducers and their interactions. External or implant-side transmitters generate focused ultrasound beams; matching and coupling interfaces ensure efficient acoustic energy propagation through tissue; receivers capture the acoustic energy and convert it to electricity; and power management/conditioning modules rectify, regulate, and store the harvested power. This framework clarifies how the individual transmitter, receiver, and dual-mode devices discussed in the following subsections fit into a complete UPT link.

#### 3.2.1. Transmit-only ultrasound power transducers (TX)

**Figure 3.**
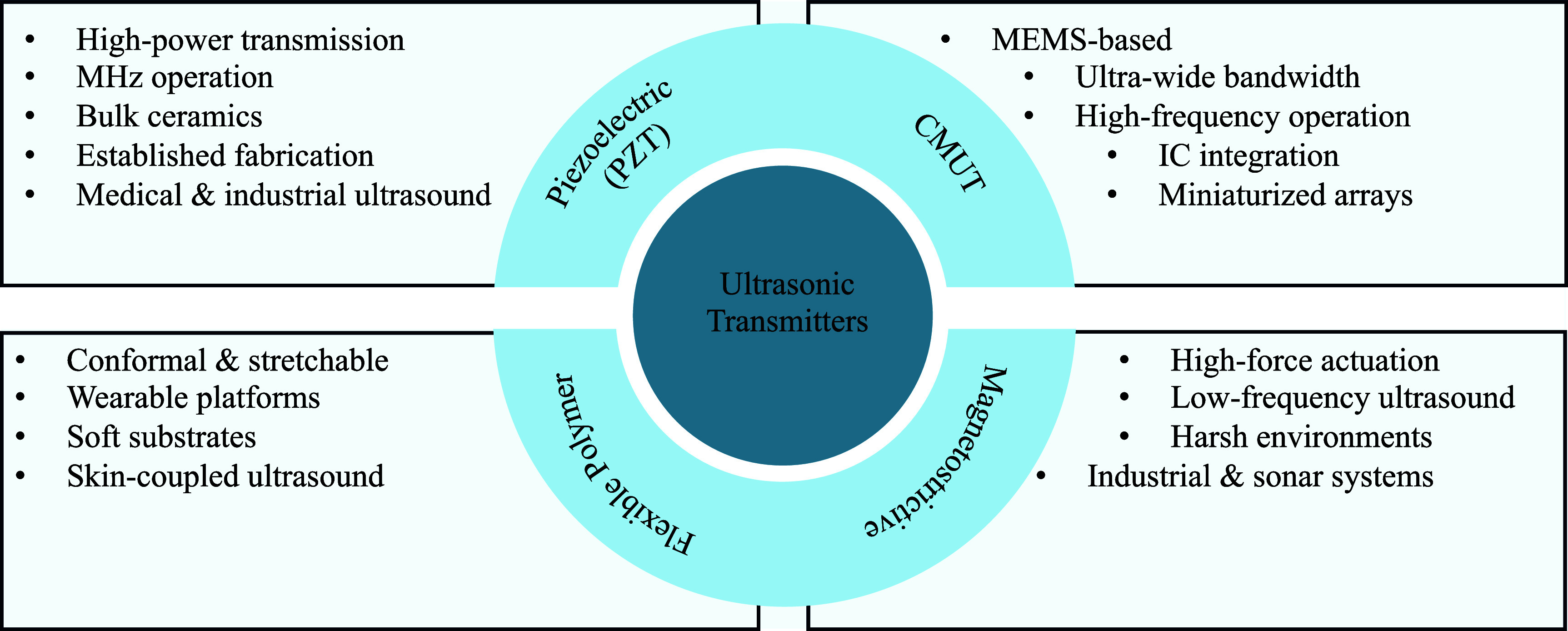
Key categories and characteristics of UPT transmitters. The schematic compares four principal transmitter types—PZT, CMUT, flexible polymer, and magnetostrictive—summarizing their main advantages, disadvantages, and representative applications for biomedical and industrial UPT systems.

Ultrasonic transmitters are specifically engineered to convert electrical signals into ultrasonic waves efficiently. The performance of transmitters is mainly determined by their electromechanical coupling efficiency, power handling capabilities, frequency response, and beam directivity. Piezoelectric ceramics (such as, PZT) represent the most common class of ultrasonic transmitters, known for their robustness and excellent energy conversion efficiency. Figure 3 provides an at-a-glance taxonomy of transmitter families, while table 3 provides the detailed side-by-side comparison of features, advantages, limitations, and representative applications.

**Table 3.** Comparison of key features, advantages, and limitations of piezoelectric, magnetostrictive, CMUT, flexible polymer transmitting transducers and PMUTs.

Transducer type	Key features	Advantages	Limitations	Common applications	References
Piezoelectric	Converts electrical energy into mechanical vibration via piezoelectric effect; ceramic-based, high electromechanical coupling	High efficiency, robust performance, broad frequency range	Narrow bandwidth, impedance matching required, lead content concerns	Medical implants, industrial sensors, ultrasonic imaging, structural monitoring	[52–54]

Magnetostrictive	Mechanical deformation in response to magnetic fields, high power capability	Robust, suitable for high power levels, durable in harsh environments	Lower efficiency, external magnetic bias required	High-power industrial applications, sonar systems, actuation devices	[47, 48]

CMUTs (Capacitive micromachined Ultrasonic transducers)	MEMS-based capacitive membranes that vibrate to generate ultrasound; integrated fabrication with electronics	Excellent miniaturization, broad bandwidth, high integration capability with ICs	Moderate efficiency, sensitivity to bias voltage requirements, limited power output	High-frequency medical imaging, precision therapeutic ultrasound, compact sensors	[39, 46]

Flexible polymer transducers	Polymer-based piezoelectric layers, mechanically flexible and stretchable	High conformability, biocompatibility, lightweight and wearable	Limited efficiency, reduced maximum power output, less mature technology	Wearable electronics, epidermal health monitors, therapeutic patches	[49]

PMUTs (Piezoelectric micromachined ultrasonic transducers)	MEMS-based ultrasonic transducers using thin-film piezoelectric layers (e.g. AlN, PZT, ScAlN) on micromachined membranes; operate via flexural vibration modes	Low driving voltage, CMOS-compatible fabrication, excellent miniaturization, good array scalability, lower power consumption than CMUTs	Lower acoustic output and bandwidth compared with bulk piezoelectric transducers, material stress and residual curvature issues, limited penetration depth	Short-range medical imaging, implantable and wearable ultrasound devices, gesture recognition, range finding, intrabody sensing	[12, 51, 55, 56]

Beyond these general performance considerations, transmitter behavior also exhibits strong technology-dependent characteristics that are not captured by efficiency metrics alone. From a transducer physics perspective, transmitter performance is governed by how efficiently a given technology converts electrical excitation into mechanical displacement under high-field drive. For piezoelectric ceramics such as PZT, high electromechanical coupling and large dielectric permittivity enable strong acoustic output and robust power handling, making them well suited for transmission. In contrast, polymer-based piezoelectrics (e.g. PVDF) exhibit lower permittivity and coupling, which limits transmit efficiency despite their mechanical flexibility. CMUT-based transmitters rely on electrostatic actuation; as a result, their transmit sensitivity scales primarily with membrane geometry and bias conditions, leading to similar efficiency trends in both transmit and receive modes. PMUTs can exhibit higher apparent transmit sensitivity than receive sensitivity in many implementations. This is because PMUTs rely on the direct piezoelectric effect in a thin-film piezoelectric layer on a flexural membrane, where only a limited active piezoelectric volume and a non-uniform strain field contribute to charge generation under acoustic loading. As a result, the effective electromechanical transduction in receive can be reduced in many designs [57, 58]. In contrast, CMUTs operate as biased capacitive transducers, where small membrane motion produces a measurable capacitance change and thus a receive current/voltage signal under DC bias, enabled by strong electrostatic transduction in submicron gaps [59, 60].

#### 3.2.2. Receive-only ultrasound energy harvesters (RX)

In contrast to transmitters, receiver performance is governed by a different set of technology-dependent sensitivities, as receivers must efficiently convert incident acoustic pressure into electrical signals under low-power and low-noise conditions. For piezoelectric receivers, sensitivity is strongly influenced by material properties such as dielectric permittivity and the piezoelectric voltage coefficient (*g*-constant). High-permittivity ceramics like PZT provide strong charge generation but relatively lower voltage sensitivity, whereas low-permittivity polymers such as PVDF exhibit higher *g*-constants and voltage sensitivity, making them attractive for sensing applications despite lower absolute harvested power. CMUT receivers show comparable scaling in transmit and receive sensitivity because both modes are governed by membrane capacitance modulation, enabling broadband and symmetric TX/RX behavior. PMUT receivers, however, generally demonstrate reduced receive sensitivity compared to their bulk piezoelectric counterparts because of the relatively low electro-mechanical coupling coefficient of flexural modes.

Ultrasonic receivers convert incident acoustic waves into electrical power. Key metrics include sensitivity, bandwidth, conversion efficiency, and achievable miniaturization. In most UPT links, receivers must capture and rectify acoustic energy at relatively low power levels. Piezoelectric receivers remain dominant because of their high sensitivity and mature fabrication. CMUT-based receivers are increasingly used for highly miniaturized, broadband implementations, while flexible polymer receivers are attractive for wearable applications due to mechanical compliance and skin compatibility.

**Figure 4.**
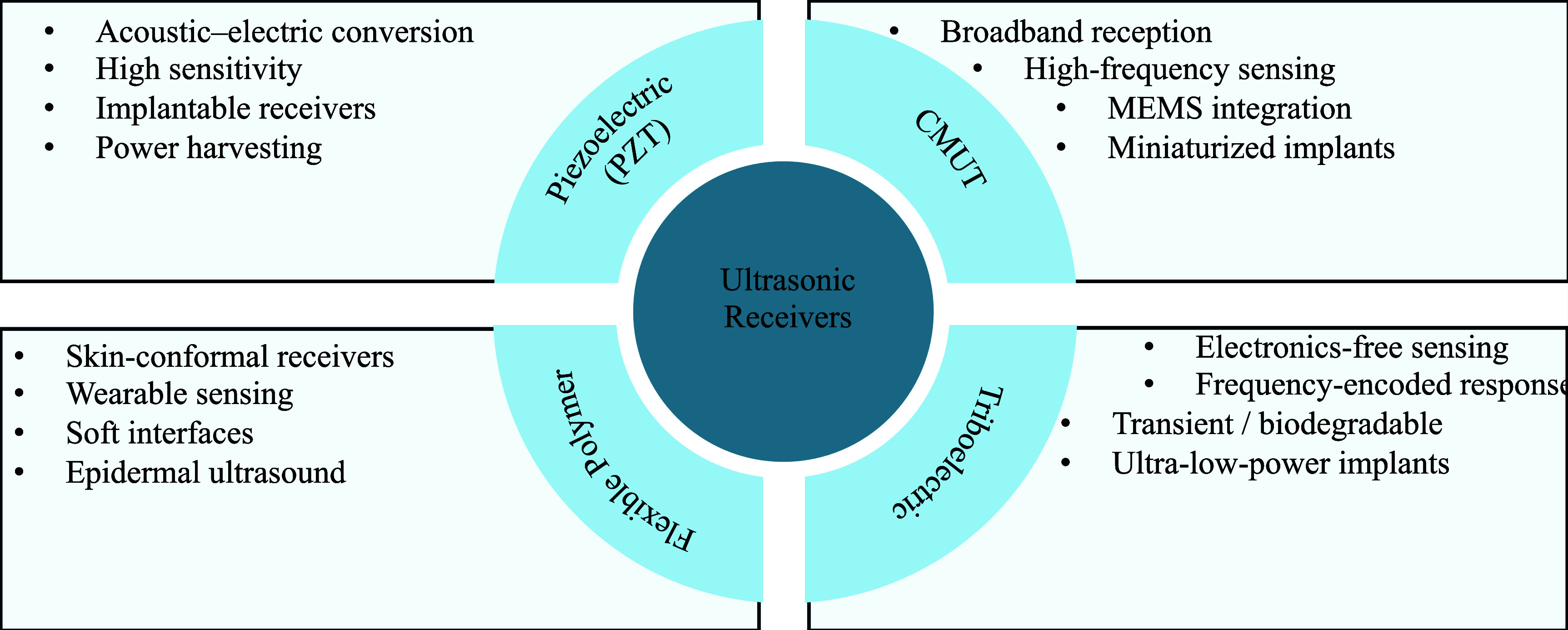
Key categories and characteristics of UPT receivers. The diagram compares four principal receiver types—PZT, CMUT, flexible polymer, and triboelectric—highlighting their major advantages, disadvantages, and representative applications in biomedical and wearable UPT systems.

To provide an at-a-glance comparison, figure 4 summarizes the major UPT receiver categories. Piezoelectric receivers provide established, high-sensitivity harvesting; CMUT receivers support broadband operation at higher frequencies; flexible polymer receivers offer mechanical conformity for wearable/epidermal use; and triboelectric receivers target ultralow-power, transient, or bioresorbable operation. This system-level view complements the detailed specifications presented in table 4.

**Table 4.** Comparison of key features, advantages, and limitations of piezoelectric, CMUT, and flexible polymer **receiving** transducers.

Transducer type	Key features	Advantages	Limitations	Common applications	References
Piezoelectric	Efficiently converts mechanical vibrations to electrical signals; ceramic-based, highly sensitive	High sensitivity, established technology, reliable power harvesting	Rigid structure, requires impedance matching, environmental concerns with lead-based materials	Implantable medical devices, wireless sensor nodes, power harvesting systems	[61–66]

CMUTs (Capacitive micromachined ultrasonic transducers)	Capacitive microstructures optimized for acoustic-to-electric energy conversion; excellent integration capabilities	High sensitivity, broadband response, effective at high frequencies	Lower output power, bias voltage requirement, complexity of fabrication	Miniaturized biomedical implants, precision diagnostics, integrated sensing platforms	[39, 67]

Flexible polymer transducers	Soft, flexible piezoelectric polymers optimized for acoustic reception	Excellent mechanical conformability, biocompatible, suitable for skin applications	Lower efficiency and sensitivity compared to ceramic-based transducers, limited maximum power output	Epidermal sensing, wearable medical monitoring, flexible ultrasound detection systems	[50]

Triboelectric	Ultra-wideband (∼20 kHz–10 MHz); soft films or injectable/biodegradable variants	Detuning-tolerant to depth/motion; conformal, lightweight; transient options (no explant)	Lower efficiency/power than piezo; high output impedance; performance can drift with fluids/surface wear	Low-power biosensing triggers; wound/antibacterial or regenerative patches; short-term/transient implants	[68, 69]

#### 3.2.3. Dual-mode ultrasound transducers for bidirectional power transfer (TX–RX)

Dual-mode transducers that both transmit and receive ultrasound are increasingly used in UPT systems. They require a trade-off between transmit efficiency and receive sensitivity. Piezoelectric and CMUT devices are common choices because their electromechanical behavior and fabrication processes integrate well with electronics [70]. Using a single element or array for both functions simplifies packaging and supports miniaturization [44]. These transducers are particularly advantageous in biomedical imaging and therapeutic applications, where precise energy transfer and responsive signal detection are both critical [45].

Beyond their ability to alternately transmit and receive, dual-mode UPT transducers also enable true bi-directional links that combine power delivery with simultaneous data communication. As discussed in section 4.5, such systems support active telemetry, passive backscatter, and resonant sensing, all built on the same Tx/Rx hardware. By leveraging the same piezoelectric or CMUT elements for both energy and information transfer, these designs reduce implant size and simplify packaging, while still allowing robust closed-loop control of power delivery and sensing functions [71].

Recent studies of ultrasonic backscatter modulation and frequency-division or time-division multiplexing further demonstrate that dual-mode UPT links can sustain continuous powering while transmitting sensor data from deep implants, strengthening their potential for next-generation bioelectronic interfaces [19, 72]. To provide a concise comparison of the most common implementations, table 5 summarizes key features, advantages, limitations, and representative applications of dual-mode ultrasonic transducers used in UPT systems. The table covers piezoelectric and CMUT devices as well as integrated bi-directional UPT links, highlighting how these transducers can operate alternately as transmitter and receiver or even support simultaneous power delivery and data communication. This overview complements the discussion above by showing how different material systems and circuit strategies balance transmit efficiency, receive sensitivity, and bidirectional energy–data functionality in compact implantable and wearable designs.

**Table 5.** Comparison of key features, advantages, and limitations of piezoelectric and CMUT **dual-mode** transducers.

Transducer type	Key features	Advantages	Limitations	Common applications	References
Piezoelectric dual-mode	Piezoelectric ceramics capable of both efficient wave emission and high sensitivity detection	Simplifies device architecture, proven reliability, wide operational frequency range	Compromise between transmit efficiency and receiver sensitivity, requires impedance matching	Medical imaging, therapeutic ultrasound, structural health monitoring	[5, 21]

CMUT dual-mode	Capacitive micromachined arrays optimized for both ultrasonic generation and sensing; integrated MEMS fabrication	Miniaturized, broad bandwidth, effective simultaneous transmit–receive operation, high integration	Lower overall power output compared to dedicated transmitters, complexity in electronics integration	Integrated biomedical imaging and therapy, advanced sensing applications	[73]

Bi-directional UPT links	Use dual-mode transducers for simultaneous power transfer + data telemetry	Enables power + communication over same acoustic channel; supports active telemetry, backscatter, or resonant sensing	Added circuit complexity; efficiency trade-off between power and data	Intrabody communication networks, battery-free implants, closed-loop therapy	

## 4. Biomedical applications of UPT

### 4.1. Neurostimulation and neuromodulation

**Figure 5.**
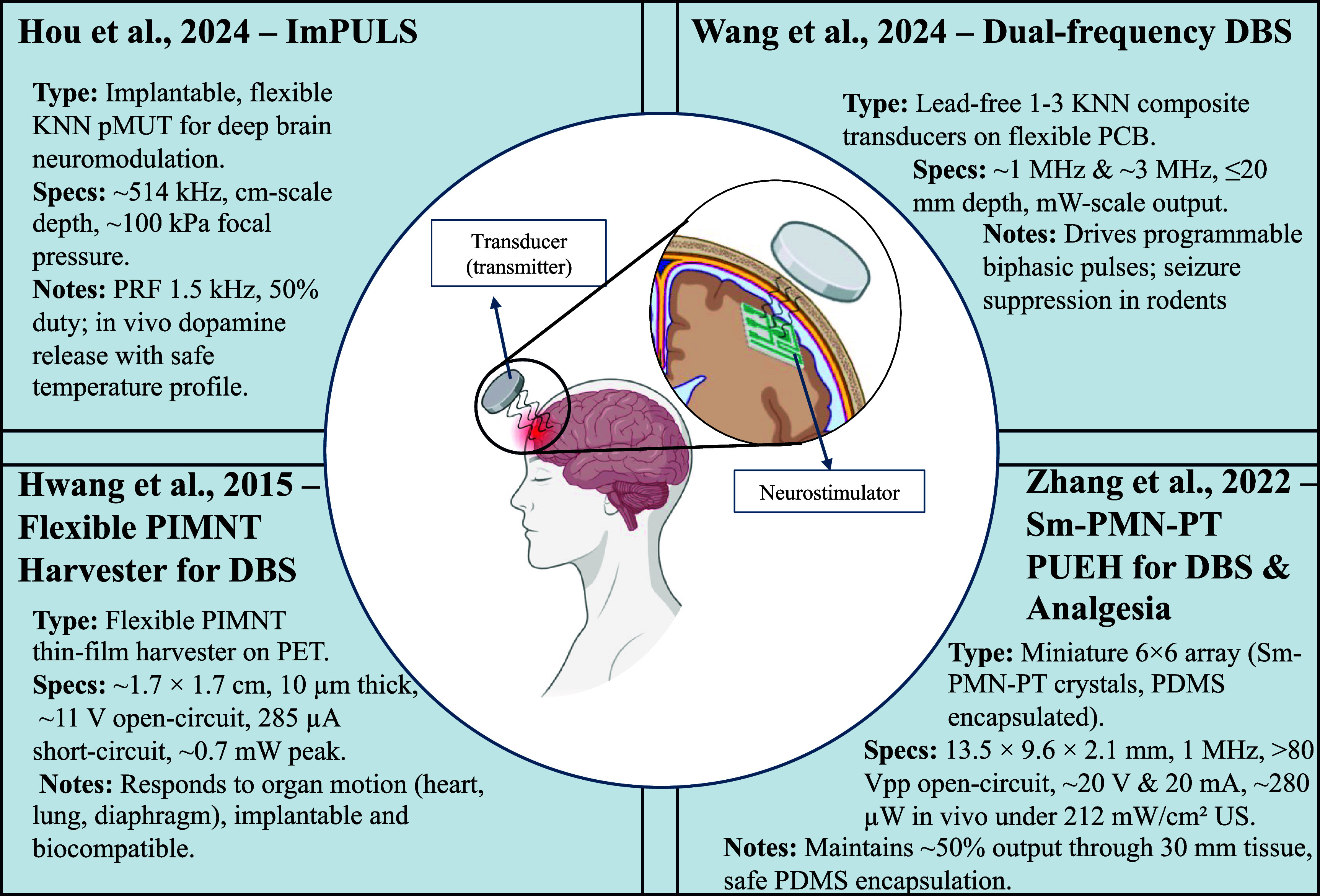
UPT for neurostimulation and neuromodulation. Representative UPT systems for neurostimulation and neuromodulation. Examples include (top left) an implantable flexible KNN pMUT for deep-brain stimulation [74], (top right) a dual-frequency lead-free KNN array on flexible PCB for programmable seizure suppression [75], (bottom left) a thin-film PIMNT energy harvester that converts organ motion to power deep-brain stimulators [76], and (bottom right) a miniature Sm-PMN-PT crystal array enabling both DBS and analgesia [77]. The central schematic highlights the wireless energy path from an external transmitter through the skull to an implanted neurostimulator.

Figure 5 highlights state-of-the-art UPT systems developed for neurostimulation and neuromodulation. These representative devices demonstrate how ultrasonic energy can be wirelessly delivered through the cranial bone to power implantable stimulators, enable dual-frequency operation, or harvest energy from endogenous organ motion. Collectively, these designs showcase the material advances, miniaturization strategies, and power-management solutions that are driving next-generation deep-brain stimulation (DBS) and neuromodulation therapies.

The first generation of ultrasonic stimulators proved that millimeter-scale implants could fire peripheral nerves without a battery. One example is the self-focused 3.6 MHz link that powered an 8 mm^3^ sciatic-nerve stimulator and delivered programmable pulse trains with centimeter-scale penetration. DBS devices soon followed: a lead-free, dual-frequency receiver built from porous 1–3 KNN composites now harvests both 1.5 MHz and 3.0 MHz acoustic energy, producing programmable biphasic pulses that suppressed epileptiform activity at 15 mm depth while the surface intensity stayed below 0.5 mW cm^−2^.

Current work adds intelligence and further miniaturization. ImPULS, a flexible potassium–sodium–niobate (KNN) PMUT with an overall footprint of only about 140 *µ*m and an active piezo element ∼100 *µ*m in diameter, focuses ∼500 kHz–1 MHz ultrasound to evoke time-locked dopamine release, drawing its entire operating power from an external transducer [74]. Zeng *et al* then demonstrated a wireless, self-adaptive spinal cord stimulator that harvests ultrasound through a thin PZT plate, feeds a convolutional neural network running on-implant, and automatically scales stimulation to rodent pain states—completely battery-free [78]. A parallel effort showed a millimeter-scale PMUT stimulator delivering 0.8 mA to peripheral nerves with 35% link efficiency through 12 mm of tissue [79]. Together, these studies confirm that centimeter-deep, lead-free and even AI-enabled neurostimulators are now practical with UPT.

### 4.2. Cardiovascular implants

**Figure 6.**
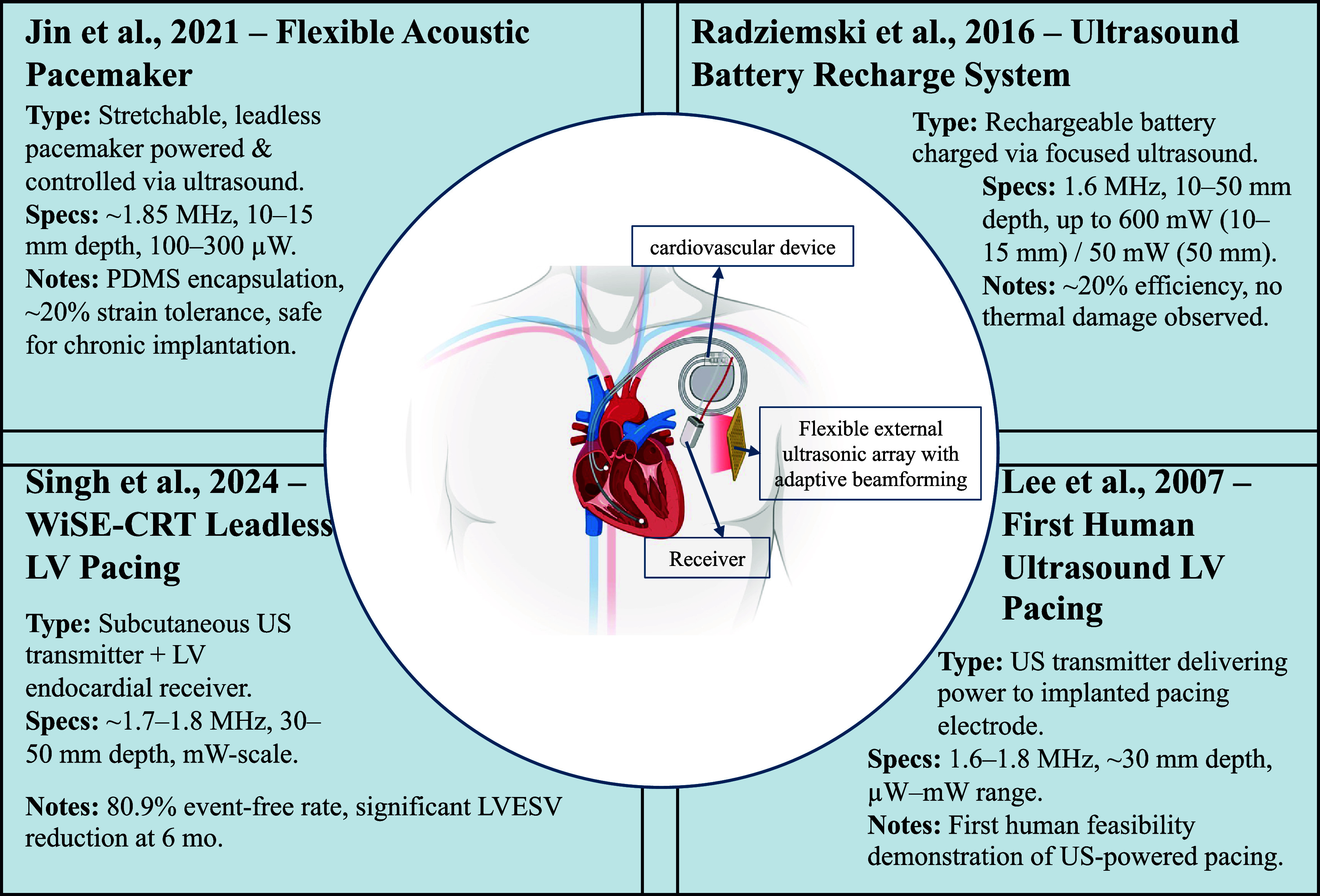
Representative ultrasonic power transfer systems for cardiovascular implants, including flexible acoustic pacemakers, leadless left-ventricular pacing systems, ultrasound-based battery recharging, and the first human demonstration of ultrasound-powered cardiac pacing. A central schematic illustrates external ultrasonic energy delivery to an implanted cardiac device.

Figure 6 highlights several representative UPT systems developed for cardiac therapy. These include flexible, leadless pacemakers powered and controlled wirelessly by ultrasound, rechargeable battery systems that leverage adaptive beamforming for efficient energy delivery, and both pre-clinical and clinical demonstrations of left-ventricular pacing powered entirely by external ultrasonic transmitters. Together these examples illustrate the rapid progress of UPT toward safe, fully implantable cardiac rhythm management. Across the cardiovascular UPT literature, authors frequently state compliance with the diagnostic MI ⩽ 1.9 criterion; however, several demonstrations operate at derated ISPTA.3 levels >720 mW cm^−2^ (non-ophthalmic), which contravenes diagnostic-imaging limits and is more relevant to thermal loading during long-duration transmissions. Consequently, ISPTA.3—rather than MI—should be treated as the primary constraint for UPT protocol design and reporting.

In addition to powering therapeutic implants, ultrasonic energy transfer has also enabled significant progress in self-powered implantable biomedical devices through innovative energy-harvesting approaches. A notable example is a MEMS-based broadband piezoelectric ultrasonic energy harvester (PUEH), which was developed to address size constraints and power fluctuation issues in implantable devices [80]. Utilizing a microfabricated array of PZT diaphragms, this device addresses critical challenges such as the large size of conventional piezoelectric transducers and power fluctuations due to standing wave effects at varying implant depths. By adjusting the input ultrasound frequency within its wide operational bandwidth, the MEMS-based PUEH effectively mitigates standing wave-induced power loss, achieving a substantial increase in power density—from 0.59 *μ*W cm^−2^ at 250 kHz to 3.75 *μ*W cm^−2^ at 240 kHz at a 1 cm distance. This adaptability is particularly beneficial for practical biomedical applications, where precise implant positioning is challenging due to anatomical variability and surgical constraints, demonstrating significant potential as a robust power source for various biomedical implants.

Clinical translation is led by the WiSE™ cardiac-resynchronization therapy (CRT) system, where a sub-pectoral phased transmitter energizes a rice-grain piezo electrode on the left-ventricular endocardium and achieved >90% capture efficacy in the SOLVE-CRT study. Complementing this human evidence, the MEMS broadband PUEH mitigates standing-wave losses by sweeping its drive frequency and boosts power density from 0.59 *µ*W cm^−2^ to 3.75 *µ*W cm^−2^ at 1 cm depth [54].

Adaptive beamforming now adds positional freedom. Li *et al* built a 16-element phased array (1 cm × 1 cm) that steers a 680 kHz beam toward a 2 mm cube receiver; the implant delivered ≈0.9 mW after rectification at 10 mm water path and ≈0.16 mW at 5 mm, all while compensating transmitter-to-receiver misalignment through real-time phase control. The same link carried a 1 kbit s^−1^ backscatter telemetry channel over 4 cm, demonstrating simultaneous power and data transfer for next-generation active cardiac implants [53]. Although these results were obtained in water, the centimeter-scale range and adaptive focusing directly address the alignment and depth challenges posed by intrathoracic pacing leads.

### 4.3. Drug delivery and therapeutic microdevices

**Figure 7.**
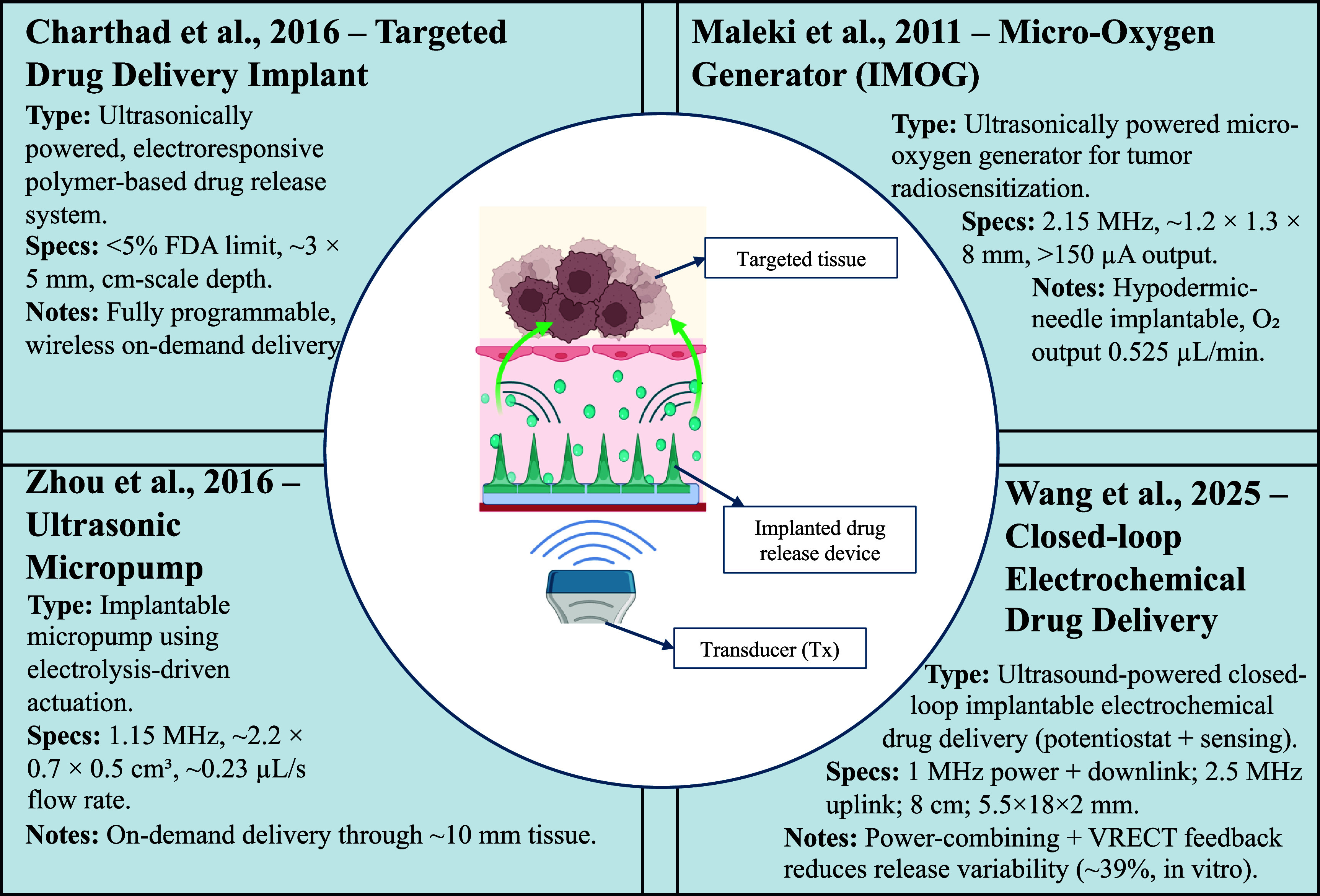
UPT-enabled implantable therapeutic delivery systems. Representative UPT strategies for targeted therapy are shown: (top left) an electroresponsive polymer-based drug delivery implant for wireless, on-demand release [81]; (bottom left) an ultrasonic micropump using electrolysis-driven actuation [82]; (top right) a micro-oxygen generator (IMOG) for tumor radiosensitization [83]; and (bottom right) a wireless implantable closed-loop electrochemical drug delivery system that integrates ultrasound power/communication with rectifier-voltage feedback to improve dosing consistency [84]. The central schematic illustrates acoustic energy transfer from an external transducer to an implanted drug-release device for noninvasive, programmable therapy.

Figure 7 summarizes representative UPT strategies for programmable drug delivery and therapeutic microdevices. Focused ultrasound can power miniature pumps, electroresponsive reservoirs, and oxygen-generation modules to enable controlled dosing at clinically relevant depths. Together, these examples illustrate UPT’s potential for minimally invasive, patient-tailored therapy.

Ultrasonically powered implants excel at releasing therapeutics exactly where and when they are needed. A flagship example is the implantable micro-oxygen generator (IMOG), which rectifies a 2.15 MHz acoustic link to drive water electrolysis [83]. The needle-deployable capsule (1.2 mm × 1.3 mm × 8 mm) produces ≈0.5 ml min^−1^ of O₂, restoring normoxia in pancreatic-tumor xenografts in <10 min and thereby boosting the efficacy of radiotherapy. However, several works emphasize MI while omitting derated ISPTA.3 and receiver directivity. Because therapeutic bursts can be long and receivers can be highly directional, authors should report ISPTA.3 with duty cycle/beam area and the receiver’s acceptance angle (−3 dB) to contextualize heating and targeting.

An ultrasound-powered tumor treating device (UP-TTD) generated localized electromagnetic pulses that disrupted mitotic activity in brain tumors, reducing tumor area by 78% *in vivo* [85]. The system’s tunable stimulation profile was achieved without enlarging the implant footprint, thanks to wireless ultrasound actuation. This kind of wireless, miniaturized implant is emblematic of what UPT enables: localized treatment, reduced invasiveness, and improved safety.

### 4.4. Tissue repair and regenerative implants

**Figure 8.**
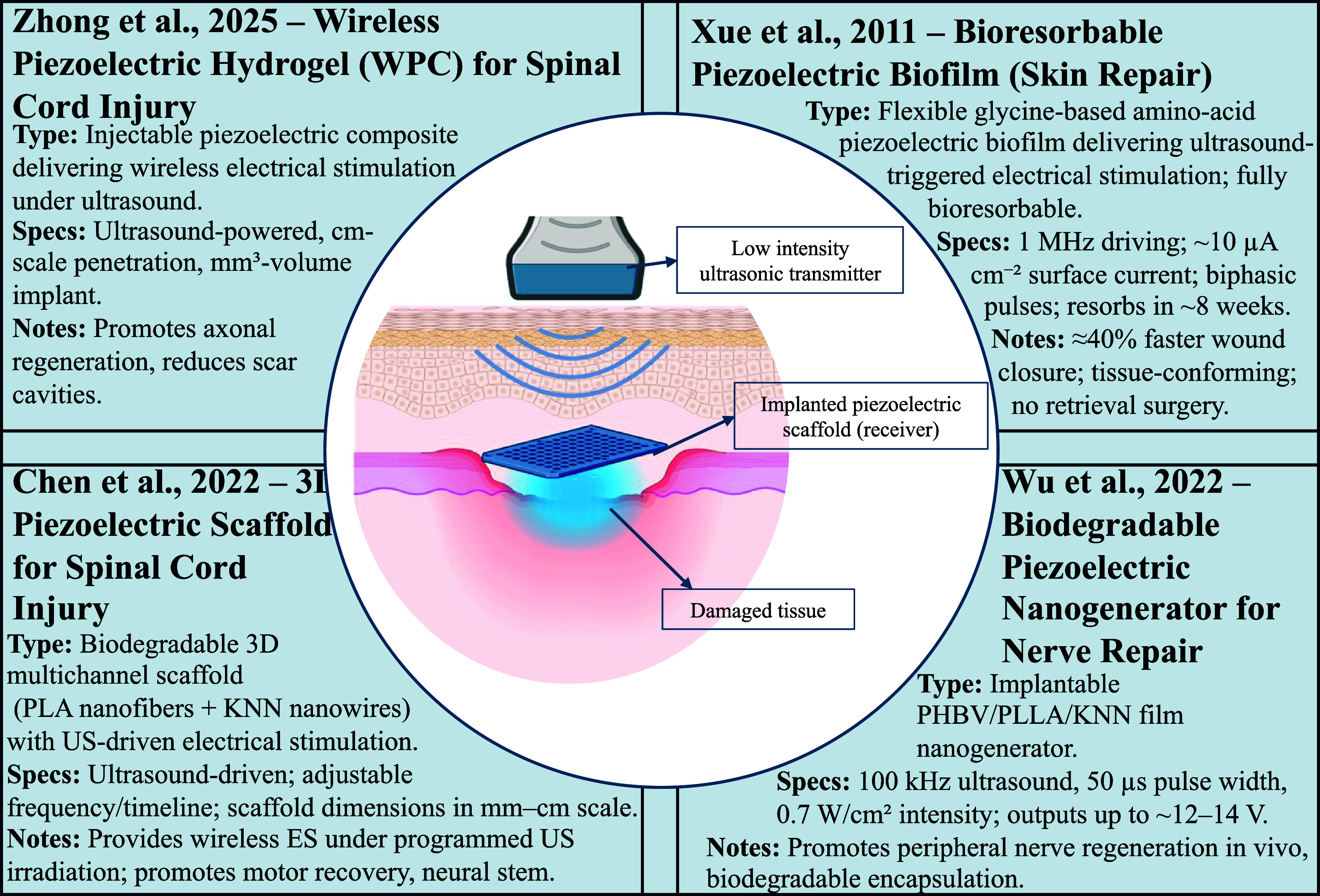
UPT for tissue repair and regenerative implants. Representative UPT systems for tissue repair and regenerative implants. Shown are (top left) an injectable wireless piezoelectric hydrogel (WPC) that delivers ultrasound-triggered electrical stimulation (ES) to promote axonal regeneration in spinal cord injury [86]; (bottom left) a 3D biodegradable piezoelectric scaffold enabling frequency-programmable, ultrasound-driven stimulation for spinal cord repair [87]; (top right) a flexible, bioresorbable glycine-based piezoelectric biofilm that converts 1 MHz ultrasound into low-voltage biphasic ES (∼10 *µ*A cm^−2^), shortens skin-wound closure by ∼40%, and fully resorbs by ∼8 weeks [88]; and (bottom right) a biodegradable piezoelectric nanogenerator that supports peripheral nerve regeneration [89]. The central schematic illustrates how a low-intensity external ultrasonic transmitter wirelessly drives an implanted piezoelectric scaffold at the injury site.

Figure 8 highlights state-of-the-art UPT strategies for tissue repair and regenerative medicine. These devices include injectable hydrogels and biodegradable 3D scaffolds and bioresorbable piezoelectric biofilms that deliver localized, ultrasound-triggered ES, alongside biodegradable piezo-nanogenerators designed to accelerate tissue and nerve healing. Here, ES refers to delivering controlled, charge-balanced electrical pulses to target tissue to promote/guide repair (e.g. enhancing cell migration and proliferation, angiogenesis, and neuromuscular activation while limiting inflammatory pain). In UPT devices, the incident ultrasound is converted by a piezoelectric element into low-voltage biphasic pulses that are routed to the tissue. Together they showcase the growing versatility of UPT in promoting neural repair, peripheral nerve regeneration, and enhanced tissue oxygenation.

Ultrasound‐activated piezoelectric scaffolds now offer on-demand ES for hard- and soft-tissue regeneration. A biodegradable piezoelectric scaffold composed of PLA and KNN nanowires delivers programmable electrical cues when triggered by ultrasound, promoting spinal cord repair through enhanced stem cell differentiation and angiogenesis [87]. Under 1 MHz ultrasound it generates tens of microamperes of alternating current, steering neural-stem-cell differentiation, angiogenesis and axonal extension after spinal-cord injury. Building on that concept, a ZnO@PCL/PVDF nanofibrous matrix reported in nano research (2024) produces biomimetic 30–80 mV potentials when insonated, accelerating osteogenesis and suppressing bacterial colonization in a rat femoral-defect model [90]. In parallel, a 3D porous piezo-hydrogel shifts macrophages toward an M2 phenotype under low-intensity pulsed ultrasound, reducing inflammation and doubling new-bone volume in critical calvarial defects [91].

Recent work extends the concept to fully bioresorbable soft patches. Xue *et al* reported a glycine-based amino-acid biofilm that is both highly piezoelectric and transient. When driven by a 1 MHz external transducer the film produces ≈10 *µ*A cm^−2^ surface current, shortens full-thickness skin-wound closure by ∼40%, and disappears completely eight weeks after implantation, eliminating the need for surgical retrieval [88].

Antibacterial regeneration has also been demonstrated. Li-doped ZnO microfiber mats convert low-intensity ultrasound (1.0 W cm^−2^, 1.0 MHz) into reactive oxygen species and endogenous electric fields; in an infected-wound model the dual action eradicated biofilms and restored healthy granulation within ten days [92]. Complementing this, Chi *et al* developed an injectable piezo-hydrogel that reconverts ultrasound energy into both sonodynamic antibacterial therapy and low-frequency ES, accelerating closure of chronic diabetic ulcers while conforming to irregular wound beds [93].

These studies—spanning rigid scaffolds, soft films and injectable hydrogels—show that ultrasound energy harvesting can power degradable electro-therapeutic platforms for bone, nerve and skin repair, ushering in a ‘treat-and-vanish’ era for regenerative implants.

### 4.5. Ultrasonic telemetry & intrabody communication

Ultrasound WPT (UPT) has evolved from purely unidirectional energy delivery into a platform that supports power-enabled telemetry, where data transmission is tightly constrained by and secondary to the power-transfer architecture. Recent studies demonstrate that a single ultrasonic link can simultaneously deliver electrical power and support information transfer, either via passive backscatter modulation [19] or via power-and-data co-designed ultrasonic transmission [16]. Importantly, the primary challenge in these systems is not achieving high data rates, but maintaining reliable power delivery under attenuation, motion, and misalignment, particularly *in vivo* [11, 22].

High-rate ultrasonic telemetry using bulk piezoelectric or LiNbO₃ pMUT arrays illustrates the upper bounds of intrabody acoustic links, showing that power transfer and communication can coexist over centimeter-scale depths without violating safety constraints [94, 95]. At the opposite end of the spectrum, ultra-low-power backscatter schemes can enable battery-free telemetry with minimal incremental energy consumption [21]. Related resonance-/mote-based approaches also support low-power identification and telemetry in implanted systems [20]. However, these methods can be more sensitive to scattering and localization challenges [96].

Collectively, these works indicate that telemetry in UPT systems should be viewed as a power-aware auxiliary function, primarily enabling system monitoring, localization, and closed-loop control, rather than as a standalone communication objective.

**Figure 9.**
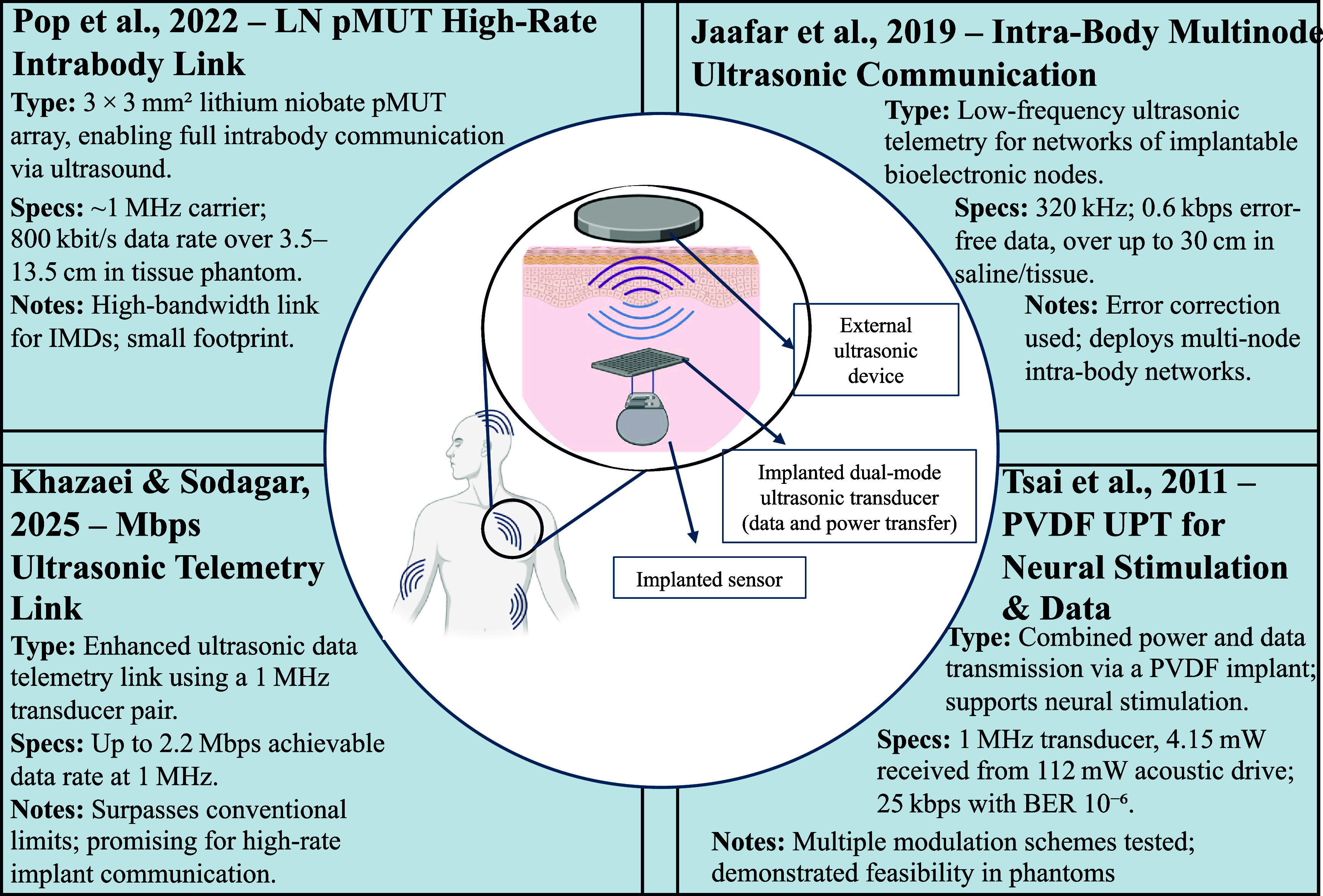
Ultrasonic telemetry and intrabody communication enabled by UPT. Representative UPT systems enabling high-rate telemetry and intrabody communication. Key demonstrations include (top left) a 3 × 3 mm^2^ lithium–niobate (LN) pMUT array providing 800 kbit s^−1^ over 3.5–13.5 cm in tissue phantoms [94]; (bottom left) a 2.2 Mbps ultrasonic telemetry link using a 1 MHz transducer pair [95]; (top right) multi-node low-frequency ultrasonic networking supporting 0.6 kbps error-free data at up to 30 cm [97]; and (bottom right) a PVDF implant that simultaneously transmits power and 25 kbps neural-stimulation data [98]. The center schematic illustrates how an external ultrasonic device energizes and communicates with an implanted dual-mode transducer and downstream sensors.

Figure 9 highlights cutting-edge ultrasonic telemetry and intrabody communication approaches that leverage UPT for simultaneous wireless power and data transfer. These systems demonstrate data rates ranging from sub-kilobit multi-node networking to multi-megabit high-bandwidth links, supporting applications from distributed biosensing to closed-loop neural stimulation.

### 4.6. Ultrasound power implant monitors

Recent progress in UPT has enabled a new class of battery-free implantable and wearable devices that operate continuously without wired connections. Although both rely on acoustic energy delivery, implantable and wearable systems face distinct power-transfer constraints and therefore occupy different design spaces.

Implantable UPT systems emphasize deep penetration, long-term stability, and minimal tissue disruption. Battery-free ultrasonic implants have demonstrated continuous physiological monitoring under full ultrasonic powering. Continuous temperature sensing has been shown using millimeter- and sub-millimeter-scale ultrasonic motes with backscatter telemetry [99, 100]. Continuous deep-tissue oxygenation monitoring has also been demonstrated with millimeter-scale ultrasonic implants [101]. These studies consistently show that power-transfer efficiency and energy-aware co-design dominate system performance and scalability for deep-tissue implants [99]. In contrast, communication bandwidth is typically not the primary bottleneck in these systems [100].

Wearable and on-skin UPT systems, by contrast, benefit from shorter transmission paths and relaxed alignment constraints. Flexible and conformal ultrasonic patches—implemented using CMUT arrays or fully lead-free piezoelectric transmitter–receiver stacks—have demonstrated efficient transcutaneous ultrasound power transfer to shallow implants or wearable electronics while improving motion tolerance through conformal acoustic coupling [102, 103]. While these systems tolerate larger form factors and higher input power, they provide a practical pathway for early clinical and consumer adoption of UPT.

From a system-level perspective, implantable devices push the limits of miniaturization and energy efficiency, whereas wearable systems explore power scalability and usability. Table 6 summarizes representative examples across these categories, highlighting how operating frequency, transducer architecture, and coupling strategy translate into practical UPT performance across applications.

**Table 6.** Representative case studies of UPT wearable devices. Key metrics include transmission frequency, penetration depth, receiver/transducer size, power efficiency, and application context. Devices span applications such as glucose monitoring, muscle activity sensing, wound healing, and cardiovascular diagnostics, demonstrating the adaptability of UPT systems in wearable and non-invasive biomedical technologies.

Medium/test model	Plant (or wearable) type:	Power transmission efficiency (PTE):	Input /output power:	Transducer parameters:					Remarks	References
				Frequency	Penetration depth:	Size	Backing	Matching layer		
On-skin wearable; *in-vitro* agarose hydrogel diffusion; *ex-vivo* porcine skin; *in-vivo* mouse tumor model	Wearable flexible ultrasound microneedle patch (wf-UMP): stretchable lead-free 1–3 mKNN ultrasonic array (4 × 6 units) + bioadhesive hydrogel elastomer couplant + dissolvable HA microneedle patch loaded with mKNN nanoparticles	N/R (therapy-focused study reports acoustic field/safety, not electrical WPT efficiency). (no numerical PTE given; safety metrics reported instead)	Input: 10–120 Vpp tested; safe operatio*n* < 70 Vpp (FDA MI/ISPTA). Output: acoustic pressure increases with input; Δ*T* ≈ 3.2 °C at 20 Vpp for 30 min on agarose. (electrical output not reported.)	Center 1.2 MHz, −6 dB BW 58%.	Single 1–3 unit > 15 mm; array up to ∼100 mm (FEA).	Piezo units: 2.5 × 2.5 × 1.5 mm, arranged 4 × 6. Microneedle patch: 10 × 10 mm, 20 × 20 cones, 600 *µ*m height, 250 *µ*m base, 550 *µ*m pitch	Biocompatible silicone elastomer encapsulation (Ecoflex) with island-bridge Cu/PI interconnects	Bioadhesive hydrogel (∼1 mm thick), acoustic impedance ∼1.54 MRayl	Stretchable ∼60%; robust skin adhesion >500 J · m^−2^; withstands ∼5 N pull; adheres under sweat; safety maintained below FDA limits; demonstrates *in-vivo* efficacy and synergy with anti-PD-1; minimal thermal rise at 20 Vpp/30 min	[104]

*In-vitro* water-tank setup (5-axis staged flat transmitter) + *ex-vivo* transcutaneous tissue demo.	Flexible 2D lead-free KNN piezoelectric receiver array (f-LFPA) for transcutaneous UETC to implants.	Not reported (paper reports output voltage/power density and SNR, but no PTE figure).	Input: within FDA ultrasound safety limits (value not specified). Output: V_oc = 22.4 V; max power density = 0.145 W cm^−2^; SNR > 30 dB	2.08 MHz (flat external transmitter, center frequency)	Not reported (*ex-vivo* transcutaneous demonstration; thickness not specified)	Piezo-unit diced to 1 mm × 1 mm × 1 mm (array built from these cubes)	Silicone elastomer Ecoflex encapsulation/backing in a sandwich device	Not specified. (No dedicated acoustic matching layer reported.)	Lead-free KNN (d₃₃ ≈ 503 pC N^−1^; d₃₃g₃₃ ≈ 2.0 × 10⁴ × 10^−1s^ m^2^ N^−1^); flexible, wide-angle receiving; simultaneous energy transfer and communication with clinical-grade SNR; operated within FDA limits; validated in water-tank and *ex-vivo* tests	[105]
*Ex-vivo* chicken-breast phantom with a PZT disk implanted at 1 cm depth; gelatin string phantom for B-mode imaging at 1–3 cm; long-term stability on TPX plate; strain tests on gelatin phantom	Patch-type 1D CMUT array (32 channels) packaged for skin attachment; rigid silicon CMUT die on flex PCB with Ca^+^-modified silk adhesive couplant and film dressing (Tegaderm)	N/R (paper reports acoustic intensity/pressure and received power; overall WPT efficiency not provided)	Drive: DC bias 70 V; 15-cycle square bursts (power) at 10.8 MHz; labeled 40 V AC in experiment figure. At focus (1 cm): pressure 65.3 kPa, intensity 156 mW cm^−2^. Received (implant): V_RMS = 2.76 mV → 166 nW (via equivalent-circuit calc; total *Z* ≈ 33.67–20i Ω)	Operational for power/data around 8.6–10.8 MHz (impulse-response 8.61 MHz, *ex-vivo* drive 10.8 MHz); −3 dB BW ≈ 85.7%; demonstrated signaling from 6–13 MHz	Measured imaging to 3 cm (SNR ⩾ ∼16.8 dB); beam-forming/focus characterized at 1–2 cm; simulation indicates usable detection up to ∼4 cm	Patch contact area 13.0 mm × 11.5 mm, thickness ∼4 mm; 32 ch linear array, 300 *µ*m pitch; each element: 336 circular cells (membrane radius 18 *µ*m, thickness 1.9 *µ*m, gap 120 nm)	None (CMUT patch packaged without a backing layer; PDMS encapsulation on device)	Ca^+^-modified silk adhesive as the coupling/matching medium (with thin PDMS electrical encapsulation)	Ultrasound-guided adaptive positioning/targeting; beam steering ±10° at 1 cm; B-mode axial/lateral resolution at 3 cm ≈ 4.26 mm/2.26 mm; bit-rate ≈ 7.04 MHz (single-pulse width 142 ns); 24 h imaging/beam stability maintained; concept supports conformal, long-term wearable powering + data to implants	[106]

Airborne (in-air) focused ultrasound; benchtop 16 × 16 phased array at 40 kHz; benchto*p* measurements at 10–30 cm; simulated/validated activation ranges up to ∼1.13 m (LEDs); demonstrations through fabrics (acoustically transparent materials)	Air-coupled wearable/tangible prototypes: haptic ring (micro-motor on hand), LED tokens/pixels, buzzers, and small motors powered at a distance; tabletop tangible tokens and a fabric bag with internal LED grid	∼0.084% end-to-end at 10 cm for a 270 Ω load (≈42 mW received vs 50 W array input)	Input (array): up to ∼50 W (≈2.5 A × 20 V) at maximum drive. Output (receiver): ≈42 mW at 10 cm (optimal ≈270 Ω load); ≈10 mW at 30 cm; activation thresholds: LED ≈ 600 Pa, buzzer ≈ 900 Pa, motor ≈ 1800 Pa	40 kHz phased-array focusing (air-coupled)	N/A (air-coupled). Practical operating distance: components activated up to ∼1.13 m (LED), ∼0.79 m (buzzer), ∼0.38 m (motor) above array	Emitter array: 16 × 16 elements; each emitter Ø ≈ 10 mm; array width ≈ 16 cm; acoustic *λ* ≈ 8.6 mm at 40 kHz. Typical receivers: 10–16 mm air-ultrasonic transducers	N/A for air-array; receivers are discrete air transducers without special backing in demos	None (air coupling); ultrasound passes through light fabrics with modest attenuation; no gel/hydrogel used	Phased-array can form multiple focal points with independently tuned amplitudes; orientation matters (large loss at steep incidence); measured received-power vs distance (≈35 mW @10 cm → ≈ 10 mW @30 cm); safety of in-air ultrasound discussed; focal SPL at 20 cm can reach high localized values while rapidly decaying outside focus	[107]

## 5. Challenges and future directions of ultrasound wireless power transmission

Despite significant advances in ultrasound wireless power transmission, several fundamental challenges continue to limit its efficiency, robustness, and scalability across applications. Addressing these issues is essential for translating laboratory demonstrations into reliable, real-world systems.

### 5.1. Propagation losses and alignment robustness

One of the most persistent challenges in UPT is efficiency loss during acoustic propagation, particularly when ultrasound travels through heterogeneous or lossy media such as biological tissue. Frequency-dependent attenuation, scattering, and impedance mismatches can substantially reduce the delivered acoustic energy, especially at increasing depths. Nevertheless, it is important to recognize that, at frequencies below approximately 10 MHz, ultrasonic attenuation in soft tissue is generally lower than electromagnetic (RF) losses, which continues to motivate UPT for implantable and intrabody applications [108].

Beyond attenuation, misalignment between transmitting and receiving transducers represents a critical system-level bottleneck. Even modest angular or lateral offsets can significantly degrade acoustic coupling and power transfer efficiency. This challenge is amplified for miniaturized implants or motes, which may be difficult to localize *in vivo* due to competing scatterers and limited imaging contrast. Future progress will likely depend on alignment-tolerant UPT architectures, including self-steering beams, adaptive phased arrays, and transducer designs that maintain efficient coupling over wider angular and positional ranges.

### 5.2. Materials, biocompatibility, and long-term reliability

Material selection remains a central concern in UPT system design, particularly for biomedical and wearable applications. Many high-performance piezoelectric materials, such as monolithic PZT piezoceramics, offer excellent electromechanical coupling but raise concerns regarding toxicity and long-term biocompatibility. Although encapsulation strategies can mitigate these risks, they introduce additional complexity and may impact acoustic performance. Additionally, for piezoelectric devices, improving energy-conversion efficiency relies on optimizing material and microstructural properties (e.g. composition/doping, domain engineering, and thickness scaling), while mechanical flexibility is achieved at the device level through thin-film implementations, composite architectures, or integration on flexible substrates with suitable encapsulation.

Emerging lead-free piezoelectrics, flexible polymers, and biodegradable composites offer promising alternatives, enabling conformal devices with improved tissue compatibility and reduced environmental impact. However, these materials often involve trade-offs among coupling efficiency, mechanical durability, and manufacturability. A key research challenge is therefore to co-optimize acoustic performance, mechanical compliance, and long-term stability, particularly under continuous or high-duty UPT operation.

### 5.3. Miniaturization versus power scalability

Miniaturization is essential for implantable and wearable UPT systems [109], yet reducing transducer dimensions inherently constrains acoustic aperture and output power. As a result, many compact receivers operate near the threshold of usable harvested power, limiting system functionality. Conversely, scaling UPT to higher power levels—relevant for industrial sensing or consumer electronics—demands transducers and interfaces capable of sustaining higher acoustic intensities without thermal or mechanical degradation.

Advances in MEMS fabrication, array-level integration, and multilayer or stacked transducer architectures provide viable pathways to address this trade-off [110]. Nonetheless, achieving scalable designs that simultaneously support high power, compact form factors, and long-term reliability remains an open challenge.

### 5.4. Emerging directions and system-level opportunities

Looking forward, several emerging approaches have the potential to reshape UPT system design. Acoustic metamaterials and engineered interfaces may enable improved wave confinement, reduced reflection losses, and enhanced energy delivery through complex media. At the system level, data-driven design strategies, including artificial intelligence and machine learning, offer new opportunities to optimize transducer geometry, operating frequency, and alignment strategies across large parameter spaces.

Equally important is the expansion of UPT beyond its current biomedical focus. Industrial monitoring in harsh or inaccessible environments, distributed sensor networks, and consumer electronics charging represent promising but underexplored application domains. Integration of UPT receivers with on-board energy storage elements, such as flexible batteries or supercapacitors, could further decouple power delivery from instantaneous alignment constraints and enhance system robustness.

In summary, UPT technologies have demonstrated compelling advantages over electromagnetic alternatives in specific operating regimes, yet their widespread adoption depends on overcoming challenges related to efficiency, alignment, materials, and scalability. Continued progress will require holistic, system-level optimization, combining advances in materials science, transducer engineering, and intelligent control. With these developments, UPT is poised to become a key enabling technology for next-generation wireless power delivery across biomedical, industrial, and consumer applications.

## 6. Conclusion

UPT represents a promising solution for wireless energy transfer, with advancements in transducer technologies driving its application in diverse fields. Its ability to deliver power efficiently through various media has made UPT particularly impactful in biomedical applications, where it enables the miniaturization of implantable devices and reduces the need for invasive procedures. Beyond healthcare, UPT’s adaptability shows potential for industrial sensors, powering underwater communication networks and autonomous vehicles, wearable devices, and consumer electronics, marking it as a versatile energy transfer technology.

While UPT has made significant strides, challenges such as efficiency losses, material limitations, and scalability must still be addressed to fully realize its potential. Overcoming these challenges will require innovative materials, optimized designs, and the integration of advanced technologies like nanostructures and machine learning.

Looking forward, UPT is set to play a key role to revolutionize wireless energy transfer technologies, providing a foundation for sustainable, efficient, and non-invasive power solutions. Its continued evolution will undoubtedly shape advancements in both emerging and established industries, offering a pathway toward more efficient and accessible wireless energy technologies.

## Data Availability

No new data were created or analysed in this study.
